# On the Solutions of a Quadratic Integral Equation of the Urysohn Type of Fractional Variable Order

**DOI:** 10.3390/e24070886

**Published:** 2022-06-27

**Authors:** Amar Benkerrouche, Mohammed Said Souid, Gani Stamov, Ivanka Stamova

**Affiliations:** 1 Department of Mathématiques, Ziane Achour University of Djelfa, Djelfa 17000, Algeria; amar.benkerrouche3@gmail.com; 2 Department of Economic Sciences, University of Tiaret, Tiaret 14035, Algeria; souimed2008@yahoo.com; 3 Department of Mathematics, University of Texas at San Antonio, San Antonio, TX 78249, USA; gani.stamov@utsa.edu

**Keywords:** fractional derivative, variable-order, fixed-point theorem, quadratic integral equation, piece-wise constant functions, uryshon-type integral equations, Ulam–Hyers stability

## Abstract

In this manuscript we introduce a quadratic integral equation of the Urysohn type of fractional variable order. The existence and uniqueness of solutions of the proposed fractional model are studied by transforming it into an integral equation of fractional constant order. The obtained new results are based on the Schauder’s fixed-point theorem and the Banach contraction principle with the help of piece-wise constant functions. Although the used methods are very powerful, they are not applied to the quadratic integral equation of the Urysohn type of fractional variable order. With this research we extend the applicability of these techniques to the introduced the Urysohn type model of fractional variable order. The applicability of the new results are demonstrated by providing Ulam–Hyers stability criteria and an example. Moreover, the presented results lead to future progress and expansion of the theory of fractional-order models, as well as of the concept of entropy in the framework of fractional calculus. Further, an example is constructed to demonstrate the reasonableness and effectiveness of the observed results.

## 1. Introduction

Integral equations are an important part of the field of nonlinear analysis since many problems studied using the nonlinear analysis methods are often expressed as differential or integro-differential equations and then converted to integral equations to facilitate their study [1,2,3,4,5]. In addition, integral equations are used as models of real-world processes in biology, ecology, population dynamics and medicine [6,7,8].

The study of nonlinear integral equations in general has aroused great interest for researchers during the last two centuries, and in particular the Uryshon-type (or Friedholm-type) integral equations, which appeared in many applied problems. These equations are defined as

(1) $$y\left(t\right)=g\left(t\right)+\int_{0}^{T}K\left(t,s,y\left(s\right)\right)ds,$$

where T>0 and *g*, *K* are given functions. For more details regarding this type of equations, see [5,9].

In recent years, we can find many applications of integral and differential equations of fractional order in physics, electricity, mechanics, engineering, economics and biology (see [10,11,12,13,14,15,16]). In addition, the concept of entropy in the framework of fractional calculus has attracted research interest recently. See, for example, ref. [17] and the references therein. However, this new concept is not sufficiently developed. Any new contribution in the area of fractional differential and integral operators can offer new opportunities for future progress and expansion of the theory of fractional-order entropies. It will also contribute to the progress of the entropy methods in applied sciences such as mathematical biology and artificial neural networks that used integral equations as modeling tools [18,19,20].

There are many papers that have studied the problem of the existence of solutions of functional integral or differential equations of the fractional constant order. We will refer to some very recent publications [21,22] and the references therein. On the contrary, only a few papers have investigated the existence of solutions of such equations of fractional variable order. For example, the authors in [23] applied the Darbo’s fixed point theorem combined with Kuratowski measure of noncompactness to analyze the existence properties of a Riemann–Liouville fractional differential equation of variable order. The existence and Ulam–Hyers stability for a variable-order Caputo-type fractional differential equation have been investigated in [24]. The existence, uniqueness and stability of solutions of a Hadamard-type fractional differential equations of variable fractional order are established in [25]. A study of the existence and uniqueness of solutions of a class of Hadamard fractional differential equations of variable order has been proposed in [26]. The authors applied piece-wise constant functions, the Krasnoselskii fixed-point theorem and the Banach contraction principle. A Caputo fractional differential equation of variable order is studied in [27] and criteria for the existence of its solutions are proposed. Some new criteria for the existence and stability of solutions of a Hadamard-type fractional differential equations of variable order have been examined in [28].

However, similar research for the Urysoh-type equations is not presented in the existing literature. This is the main motivation for our study which makes the results on the topic interesting and worthwhile. Given the importance of such equations for the theory and applications, it is meaningful to consider their extension to the fractional variable order and investigate their fundamental and qualitative properties. In fact, considering the variable fractional order is challenging since the research of such types of fractional models is still in its infancy and their properties are different from the corresponding properties of systems with a constant fractional order, including the semigroup property.

Stimulated by the above discussion, in this study, we introduce a quadratic integral equation of the Urysohn type of fractional variable order of the following type

(2) $$y\left(t\right)=g\left(t\right)+\left(\Phi y\right)\left(t\right)\int_{0}^{t}\frac{1}{\Gamma (\eta (t))}\frac{u(t,s,y(s))}{{\left(t-s\right)}^{1-\eta (t)}}ds,\;\;\;t\in J:=\left[0,T\right],$$

where g:J→R is a continuous function, $u:J^{2}\times R\to R$ is a given function, Γ is the Gamma function, 1<η(t)≤2 and Φ:C(J,R)→C(J,R) is a proper operator. In the above, the notation C(J,R) represents a Banach space of all continuous functions y:J→R with the norm

∥y∥=sup{|y(t)|:t∈J}.

To the introduced new model (2) of fractional variable order, we will apply the Schauder’s fixed-point theorem and the Banach contraction principle to investigate the existence and uniqueness of its solutions. In fact, although both methods are very powerful, they are not applied in the study of the fundamental properties of the quadratic integral equation of the Urysohn type to the fractional variable order (2).

The main contributions of our research are:

1. We generalize and extend the existing quadratic integral equation of Urysohn type to the fractional variable order in the form of a piece-wise constant function;
2. Efficient existence and uniqueness criteria for the extended model are proposed;
3. The obtained fundamental results are applied in the study of the Ulam–Hyers stability of the solution;
4. An example is elaborated to demonstrate our results.

The Riemann–Liouville fractional integral approach of variable-order is adopted in our research. The manuscript is organized according to the following plan. Some definitions and properties of the Riemann–Liouville fractional integral of variable fractional order are stated in Section 2. The concepts of generalized interval, partition and piece-wise constant functions are also defined. Section 3 is devoted to our main existence and uniqueness results for the introduced integral equation of the Urysohn type of fractional variable order. Three theorems are proved by the use of the Schauder’s fixed point theorem and the Banach contraction principle. In Section 4, in order to demonstrate the applicability of the proposed existence and uniqueness results, the Ulam–Hyers stability of the solution is considered. In Section 5 an example is derived to demonstrate the new results for the proposed integral model of fractional variable order. Section 6 presents our concluding remarks and future directions.

## 2. Preliminaries

In this section, we introduce notations, definitions, and preliminary facts that are used throughout this paper.

We consider the mapping η(t):J→(1,2]. Then, the left Riemann–Liouville fractional integral (RLFI) of variable-order η(t) for a function *h* is defined as [29,30,31]

(3) $$I_{0^{+}}^{\eta (t)}h\left(t\right)=\int_{0}^{t}\frac{{\left(t-s\right)}^{\eta (t)-1}}{\Gamma (\eta (t))}h\left(s\right)ds,\;\;t>0.$$

In the case when η(t) is a constant, then RLFI coincides with the classical Riemann–Liouville fractional integral of a constant order, see, e.g., [29,30,32].

**Remark** **1 ([33,34]).** *Note that, the semigroup property does not hold for arbitrary functions η(t), v(t), i.e., in general*

$$I_{a^{+}}^{\eta (t)}I_{a^{+}}^{v(t)}h\left(t\right)\neq I_{a^{+}}^{\eta (t)+v(t)}h\left(t\right).$$

**Lemma** **1 ([35]).** *If η∈C(J,(1,2]), then:*

*(a)*  *$I_{0^{+}}^{\eta (t)}h\left(t\right)\in C\left(J,R\right)$ for h∈C(J,R);*
*(b)*  *The variable order fractional integral $I_{0^{+}}^{\eta (t)}h\left(t\right)$ exists at any point on J for $h\in C_{\delta }\left(J,R\right)=\left\{h\left(t\right)\in C\left(J,R\right),\;t^{\delta }h\left(t\right)\in C\left(J,R\right),0\leq \delta \leq 1\right\}.$*

Let J⊂R.

**Definition** **1 ([36,37]).** *The interval J will be called a generalized interval if it is either Ø, or $\left\{a_{1}\right\}$ or an interval.*

**Definition** **2 ([36,37]).** *A partition of J is a finite set P such that each x in J lies in exactly one of the generalized intervals E in P.*

**Definition** **3 ([36,37]).** *A function g:J→R is called piece-wise constant with respect to the partition P of J if for any E∈P, g is constant on E.*

In the proof of our main results we will also use the following Schauder fixed point theorem.

**Theorem** **1 ([32]).** *Assume that E is a Banach space and Λ is a convex subset of E. If F:Λ⟶Λ is compact and continuous map, then F has at least one fixed point in Λ.*

**Definition** **4.** *The Equation (2) is Ulam–Hyers stable if there exists $c_{u}>0$ such that for any ϵ>0 and for every solution z∈C(J,R) of the inequality*

(4) $$|z\left(t\right)-g\left(t\right)-\left(\Phi z\right)\left(t\right)\int_{0}^{t}\frac{1}{\Gamma (\eta (t))}\frac{u(t,s,z(s))}{{\left(t-s\right)}^{1-\eta (t)}}ds|\leq \epsilon ,\;\;t\in J$$

*there exists a solution y∈C(J,R) of Equation (2) with*

$$\left|z\left(t\right)-y\left(t\right)\right|\leq c_{u}\epsilon ,\;\;t\in J.$$

## 3. Main Existence and Uniqueness Results

We will prove our existence and uniqueness criteria under the following assumption:

**(A1)**  For n∈N there exists a partition of the interval *J* defined as
  $$P=\{J_{1}:=\left[0,T_{1}\right],J_{2}:=\left(T_{1},T_{2}\right],J_{3}:=\left(T_{2},T_{3}\right],\ldots ,J_{n}:=\left(T_{n-1},T\right]\}$$
  and a piece-wise constant function η(t):J→(1,2] with respect to P such that

$$\eta \left(t\right)=\sum_{m=1}^{n}\eta_{m}I_{m}\left(t\right)=\left\{\begin{array}{c} \eta_{1},\;\;if\;t\in J_{1},\\ \eta_{2},\;\;if\;t\in J_{2},\\ \;\;\;.\;\;\;\;\;\;\;\\ \;\;\;.\;\;\;\;\;\;\;\\ \;\;\;.\;\;\;\;\;\;\;\\ \eta_{n},\;\;if\;t\in J_{n}, \end{array}\right.$$

where $1<\eta_{m}\leq 2$ are constants, and $I_{m}$ is the index of the interval $J_{m}:=\left(T_{m-1},T_{m}\right],\;m=1,2,\ldots ,n$, (with $T_{0}=0,\;T_{n}=T$) defined as

$$I_{m}\left(t\right)=\left\{\begin{array}{c} 1,\;\;for\;t\in J_{m},\\ 0,\;\;for\;elsewhere. \end{array}\right.$$

The symbol $E_{m}=C\left(J_{m},\;R\right)$ indicates the Banach space of all continuous functions $y:J_{m}\to R$ with the norm

$${∥y∥}_{E_{m}}=\underset{t\in J_{m}}{\sup}\left|y\left(t\right)\right|,$$

where m∈{1,2,…,n}.

We will first analyze the equation defined in (2). For any $t\in J_{m},$m=1,2,…,n, the RLFI of variable order η(t) for the function $u\left(t,s,y\left(s\right)\right)\in C\left(J^{2}\times R,R\right)$, defined by (3), could be presented as a sum of left Riemann–Liouville fractional integrals of constant-orders $\eta_{m},$m=1,2,…,n.

(5) $$I_{0^{+}}^{\eta (t)}u\left(t,s,y\left(t\right)\right)=\sum_{i=1}^{i=m-1}\int_{T_{i-1}}^{T_{i}}\frac{{\left(t-s\right)}^{\eta_{i}-1}}{\Gamma (\eta_{i})}u\left(t,s,y\left(s\right)\right)ds+\int_{T_{m-1}}^{t}\frac{{\left(t-s\right)}^{\eta_{m}-1}}{\Gamma (\eta_{m})}u\left(t,s,y\left(s\right)\right)ds.$$

Thus, according to (5), for any $t\in J_{m},\;m=1,2,\ldots ,n$, Equation (2) can be written as

(6) $$y\left(t\right)=g\left(t\right)+\left(\Phi y\right)\left(t\right)\left(\sum_{i=1}^{i=m-1}\int_{T_{i-1}}^{T_{i}}\frac{1}{\Gamma (\eta_{i})}\frac{u(t,s,y(s))}{{\left(t-s\right)}^{1-\eta_{i}}}ds+\int_{T_{m-1}}^{t}\frac{1}{\Gamma (\eta_{m})}\frac{u(t,s,y(s))}{{\left(t-s\right)}^{1-\eta_{m}}}ds\right).$$

Let the function $y\in C(J_{m},R)$ be a solution of the integral Equation (6) such that y(t)≡0 on $t\in [0,T_{m-1}]$. Then, (6) is reduced to

(7) $$y\left(t\right)=g\left(t\right)+\left(\Phi y\right)\left(t\right)\left(\int_{T_{m-1}}^{t}\frac{1}{\Gamma (\eta_{m})}\frac{u(t,s,y(s))}{{\left(t-s\right)}^{1-\eta_{m}}}ds\right),\;t\in J_{m}.$$

Now, we will study Equation (7) assuming that for all m∈{1,2,…,n} the following assumptions are satisfied:

**(A2)**  There exists $K_{m}>0$ such that
  $$|\left(\Phi y\right)\left(t\right)-\left(\Phi \bar{y}\right)\left(t\right)|\leq K_{m}\left|y\left(t\right)-\bar{y}\left(t\right)\right|$$
  for each $y,\bar{y}\in E_{m}$ and $t\in J_{m}$.
**(A3)**  There exist non-negative constants α and β such that
  |(Φy)(t)|≤α+β|y(t)|
  for each $y\in E_{m}$ and $t\in J_{m}$.
**(A4)**  The function $u:J_{m}^{2}\times R\to R$ is continuous on $J_{m}^{2}\times R$ and nondecreasing with respect to its three variables, separately, and there exist constants 0≤δ≤1, $D_{m}>0$ such that
  $$t^{\delta }|u\left(t,s,y\right)-u\left(t,s,\bar{y}\right)|\leq D_{m}\left|y-\bar{y}\right|$$
  for all $\left(t,s\right)\in J_{m}^{2}$ and $y,\bar{y}\in R$.
**(A5)**  There exist a constant 0≤δ≤1 and continuous nondecreasing functions $\vartheta \in C(R_{+},R_{+})$ and $\varphi \in C(J,R_{+})$ such that for each $\left(t,s\right)\in J_{m}^{2}$ and y∈R we have
  $$t^{\delta }\left|u\left(t,s,y\right)\right|\leq \varphi \left(s\right)\vartheta \left(|y|\right).$$

Our first existence result is based on Theorem 1.

**Theorem** **2.** *Suppose that m∈{1,2,…,n}, assumptions (A1)–(A5) Nare satisfied, and there exists a constant $R_{m}$, such that*

(8) $$\frac{R_{m}}{g^{⋆}+\frac{\left(T_{m}^{1-\delta }-T_{m-1}^{1-\delta }\right){\left(T_{m}-T_{m-1}\right)}^{\eta_{m}-1}}{\left(1-\delta \right)\Gamma \left(\eta_{m}\right)}\left(\alpha +\beta R_{m}\right)\vartheta \left(R_{m}\right)\varphi^{⋆}}>1,$$

*where $\varphi^{⋆}=sup\left\{\varphi \left(s\right):s\in J_{m}\right\}$ and $g^{⋆}=sup\left\{g\left(t\right):t\in J_{m}\right\}.$*

*Then, Equation (7) has at least one solution in $E_{m}$.*

**Proof.**  Consider the operator

$$S:E_{m}\to E_{m}$$

defined by

$$\left(Sy\right)\left(t\right)=g\left(t\right)+\left(\Phi y\right)\left(t\right)\int_{T_{m-1}}^{t}\frac{1}{\Gamma (\eta_{m})}\frac{u(t,s,y(s))}{{\left(t-s\right)}^{1-\eta_{m}}}ds.$$

Define the set

$$B_{R_{m}}=\{y\in E_{m}{:∥y∥}_{E_{m}}\leq R_{m}\}.$$

Clearly, $B_{R_{m}}$ is nonempty, bounded, closed and convex subset of $E_{m}$.

Now, we will demonstrate that *S* satisfies the assumptions of Theorem 1.

STEP 1: Claim: $S\left(B_{R_{m}}\right)\subseteq \left(B_{R_{m}}\right).$

For $y\in B_{R_{m}}$, we have

$$\begin{array}{ccc} \left|(Sy)(t)\right| & \leq & \left|g\left(t\right)\right|+\left|\left(\Phi y\right)\left(t\right)\right|\int_{T_{m-1}}^{t}\frac{1}{\Gamma (\eta_{m})}\left|\frac{u(t,s,y(s))}{{\left(t-s\right)}^{1-\eta_{m}}}\right|ds\\ & \leq & \left|g\left(t\right)|+(\alpha +\beta |y\left(t\right)|\right)\int_{T_{m-1}}^{t}\frac{1}{\Gamma (\eta_{m})}\frac{1}{{\left(t-s\right)}^{1-\eta_{m}}}s^{-\delta }\varphi \left(s\right)\vartheta \left(|y\left(s\right)|\right)ds\\ & \leq & g^{⋆}+\frac{\left(t^{1-\delta }-T_{m-1}^{1-\delta }\right){\left(t-T_{m-1}\right)}^{\eta_{m}-1}}{\left(1-\delta \right)\Gamma \left(\eta_{m}\right)}{(\alpha +\beta ∥y∥}_{E_{m}}{)\vartheta (∥y∥}_{E_{m}})\varphi^{⋆}\\ & \leq & g^{⋆}+\frac{\left(T_{m}^{1-\delta }-T_{m-1}^{1-\delta }\right){\left(T_{m}-T_{m-1}\right)}^{\eta_{m}-1}}{\left(1-\delta \right)\Gamma \left(\eta_{m}\right)}{(\alpha +\beta ∥y∥}_{E_{m}}{)\vartheta (∥y∥}_{E_{m}})\varphi^{⋆}\\ & \leq & R_{m}, \end{array}$$

which means that $S\left(B_{R_{m}}\right)\subseteq B_{R_{m}}$.

STEP 2: Claim: *S* is continuous.

We presume that the sequence $\left(y_{n}\right)$ converges to *y* in $E_{m}$ and $t\in J_{m}$. Then,

$$|\left(Sy_{n}\right)\left(t\right)-\left(Sy\right)\left(t\right)|$$

$$\begin{array}{ccc} & \leq & |\left(\Phi y_{n}\right)\left(t\right)\int_{T_{m-1}}^{t}\frac{1}{\Gamma (\eta_{m})}\frac{u(t,s,y(s))}{{\left(t-s\right)}^{1-\eta_{m}}}ds-\left(\Phi y\right)\left(t\right)\int_{T_{m-1}}^{t}\frac{1}{\Gamma (\eta_{m})}\left|\frac{u(t,s,y(s))}{{\left(t-s\right)}^{1-\eta_{m}}}ds\right|\\ & \leq & |\left(\Phi y_{n}\right)\left(t\right)\int_{T_{m-1}}^{t}\frac{1}{\Gamma (\eta_{m})}\frac{u(t,s,y(s))}{{\left(t-s\right)}^{1-\eta_{m}}}ds-\left(\Phi y_{n}\right)\left(t\right)\int_{T_{m-1}}^{t}\frac{1}{\Gamma (\eta_{m})}\left|\frac{u(t,s,y(s))}{{\left(t-s\right)}^{1-\eta_{m}}}ds\right|\\ & + & |\left(\Phi y_{n}\right)\left(t\right)\int_{T_{m-1}}^{t}\frac{1}{\Gamma (\eta_{m})}|\frac{u(t,s,y(s))}{{\left(t-s\right)}^{1-\eta_{m}}}ds-\left(\Phi y\right)\left(t\right)\int_{T_{m-1}}^{t}\frac{1}{\Gamma (\eta_{m})}\left|\frac{u(t,s,y(s))}{{\left(t-s\right)}^{1-\eta_{m}}}ds\right|\\ & \leq & |\left(\Phi y_{n}\right)\left(t\right)|\int_{T_{m-1}}^{t}\frac{1}{\Gamma (\eta_{m})}\frac{1}{{\left(t-s\right)}^{1-\eta_{m}}}\left|u\left(t,s,y_{n}\left(s\right)\right)ds-u\left(t,s,y\left(s\right)\right)\right|ds\\ & + & |\left(\Phi y_{n}\right)\left(t\right)-\left(\Phi y\right)\left(t\right)|\int_{T_{m-1}}^{t}\frac{1}{\Gamma (\eta_{m})}\left|\frac{u(t,s,y(s))}{{\left(t-s\right)}^{1-\eta_{m}}}ds\right|\\ & \leq & \left(\alpha +\beta \right|y_{n}\left(t\right)|)D_{m}\int_{T_{m-1}}^{t}\frac{1}{\Gamma (\eta_{m})}\frac{1}{{\left(t-s\right)}^{1-\eta_{m}}}s^{-\delta }\left|y_{n}\left(s\right)-y\left(s\right)\right|ds\\ & + & K_{m}|y_{n}\left(s\right)-y\left(s\right)|\int_{T_{m-1}}^{t}\frac{1}{\Gamma (\eta_{m})}\frac{1}{{\left(t-s\right)}^{1-\eta_{m}}}s^{-\delta }\varphi \left(s\right)\vartheta \left(|y\left(s\right)|\right)ds\\ & \leq & [(D_{m}\left(\alpha +\beta R_{m}\right)+\varphi^{⋆}K_{m}\vartheta \left(R_{m}\right))\frac{\left(T_{m}^{1-\delta }-T_{m-1}^{1-\delta }\right){\left(T_{m}-T_{m-1}\right)}^{\eta_{m}-1}}{\left(1-\delta \right)\Gamma \left(\eta_{m}\right)}]{∥y_{n}-y∥}_{E_{m}}, \end{array}$$

i.e., we obtain

$$∥\left(Sy_{n}\right)-\left(Sy\right){∥}_{E_{m}}\to 0\;\;\;as\;\;\;n\to \infty .$$

The above relation shows that the operator *S* is continuous on $E_{m}$.

STEP 3: Claim: *S* is compact.

In order to show that *S* is compact it is enough to prove that $S(B_{R_{m}})$ is relatively compact. By Step 1, we have that $S(B_{R_{m}})$ is uniformly bounded or $S\left(B_{R_{m}}\right)=\left\{S\left(y\right):y\in B_{R_{m}}\right\}\subset B_{R_{m}}$. Thus, for each $y\in B_{R_{m}}$ we have ${∥S\left(y\right)∥}_{E_{m}}\leq R_{m}$ which means that $S(B_{R_{m}})$ is bounded. It remains to indicate that $S(B_{R_{m}})$ is equicontinuous.

For $t_{1},t_{2}\in J_{m},\;t_{1}<t_{2}$ and $y\in B_{R_{m}}$, we have

$$|\left(Sy\right)\left(t_{2}\right)-\left(Sy\right)\left(t_{1}\right)|$$

$$\begin{array}{ccc} & & \leq |g\left(t_{2}\right)-g\left(t_{1}\right)|\\ & & +|\left(\Phi y\right)\left(t_{2}\right)\int_{T_{m-1}}^{t_{2}}\frac{1}{\Gamma (\eta_{m})}\frac{u(t_{2},s,y\left(s\right))}{{\left(t_{2}-s\right)}^{1-\eta_{m}}}ds-\left(\Phi y\right)\left(t_{1}\right)\int_{T_{m-1}}^{t_{1}}\frac{1}{\Gamma (\eta_{m})}\frac{u(t_{1},s,y\left(s\right))}{{\left(t_{1}-s\right)}^{1-\eta_{m}}}ds|\\ & & \leq |g\left(t_{2}\right)-g\left(t_{1}\right)\left|+|\left(\Phi y\right)\left(t_{2}\right)\right|\int_{T_{m-1}}^{t_{2}}\frac{1}{\Gamma (\eta_{m})}\frac{u(t_{2},s,y\left(s\right))}{{\left(t_{2}-s\right)}^{1-\eta_{m}}}ds-\int_{T_{m-1}}^{t_{1}}\frac{1}{\Gamma (\eta_{m})}\frac{u(t_{1},s,y\left(s\right))}{{\left(t_{1}-s\right)}^{1-\eta_{m}}}ds|\\ & & +|\left(\left(\Phi y\right)\left(t_{2}\right)-\left(\Phi y\right)\left(t_{1}\right)\right)\int_{T_{m-1}}^{t_{1}}\frac{1}{\Gamma (\eta_{m})}\frac{u(t_{1},s,y\left(s\right))}{{\left(t_{1}-s\right)}^{1-\eta_{m}}}ds|\\ & & \leq |g\left(t_{2}\right)-g\left(t_{1}\right)\left|+\right|\left(\Phi y\right)\left(t_{2}\right)\int_{T_{m-1}}^{t_{1}}\frac{1}{\Gamma (\eta_{m})}\frac{u(t_{2},s,y\left(s\right))}{{\left(t_{2}-s\right)}^{1-\eta_{m}}}-\frac{1}{\Gamma (\eta_{m})}\frac{u(t_{1},s,y\left(s\right))}{{\left(t_{1}-s\right)}^{1-\eta_{m}}}ds|\\ & & +|\left(\Phi y\right)\left(t_{2}\right)\int_{t_{1}}^{t_{2}}\frac{1}{\Gamma (\eta_{m})}\frac{u(t_{2},s,y\left(s\right))}{{\left(t_{2}-s\right)}^{1-\eta_{m}}}ds|\\ & & +|\left(\left(\Phi y\right)\left(t_{2}\right)-\left(\Phi y\right)\left(t_{1}\right)\right)\int_{T_{m-1}}^{t_{1}}\frac{1}{\Gamma (\eta_{m})}\frac{u(t_{1},s,y\left(s\right))}{{\left(t_{1}-s\right)}^{1-\eta_{m}}}ds|\\ & & \leq |g\left(t_{2}\right)-g\left(t_{1}\right)\left|+\right|\left(\Phi y\right)\left(t_{2}\right)\int_{T_{m-1}}^{t_{1}}\frac{1}{\Gamma (\eta_{m})}\frac{u\left(t_{2},s,y\left(s\right)\right)-u\left(t_{1},s,y\left(s\right)\right)}{{\left(t_{2}-s\right)}^{1-\eta_{m}}}ds|\\ & & +|\left(\Phi y\right)\left(t_{2}\right)\int_{T_{m-1}}^{t_{1}}\frac{u(t_{1},s,y\left(s\right))}{\Gamma (\eta_{m})}\left(\frac{1}{{\left(t_{2}-s\right)}^{1-\eta_{m}}}-\frac{1}{{\left(t_{1}-s\right)}^{1-\eta_{m}}}\right)ds|\\ & & +|\left(\Phi y\right)\left(t_{2}\right)\int_{t_{1}}^{t_{2}}\frac{1}{\Gamma (\eta_{m})}\frac{u(t_{2},s,y\left(s\right))}{{\left(t_{2}-s\right)}^{1-\eta_{m}}}ds|\\ & & +|\left(\left(\Phi y\right)\left(t_{2}\right)-\left(\Phi y\right)\left(t_{1}\right)\right)\int_{T_{m-1}}^{t_{1}}\frac{1}{\Gamma (\eta_{m})}\frac{u(t_{1},s,y\left(s\right))}{{\left(t_{1}-s\right)}^{1-\eta_{m}}}ds|\\ & & \leq |g\left(t_{2}\right)-g\left(t_{1}\right)\left|+\right|\left(\Phi y\right)\left(t_{2}\right)|\int_{T_{m-1}}^{t_{2}}\frac{1}{\Gamma (\eta_{m})}\frac{|u\left(t_{2},s,y\left(s\right)\right)-u\left(t_{1},s,y\left(s\right)\right)|}{{\left(t_{2}-s\right)}^{1-\eta_{m}}}ds\\ & & +|\left(\Phi y\right)\left(t_{2}\right)||u\left(t_{1},t_{1},R_{m}\right)|\int_{T_{m-1}}^{t_{1}}\frac{1}{\Gamma (\eta_{m})}\left(\frac{1}{{\left(t_{2}-s\right)}^{1-\eta_{m}}}-\frac{1}{{\left(t_{1}-s\right)}^{1-\eta_{m}}}\right)ds\\ & & +|\left(\Phi y\right)\left(t_{2}\right)||u\left(t_{2},t_{2},R_{m}\right)|\int_{t_{1}}^{t_{2}}\frac{1}{\Gamma (\eta_{m})}\frac{1}{{\left(t_{2}-s\right)}^{1-\eta_{m}}}ds\\ & & +|\left(\Phi y\right)\left(t_{2}\right)-\left(\Phi y\right)\left(t_{1}\right)||u\left(t_{1},t_{1},R_{m}\right)|\int_{T_{m-1}}^{t_{1}}\frac{1}{\Gamma (\eta_{m})}\frac{1}{{\left(t_{1}-s\right)}^{1-\eta_{m}}}ds\\ & & \leq |g\left(t_{2}\right)-g\left(t_{1}\right)\left|+\right|\left(\Phi y\right)\left(t_{2}\right)|\int_{T_{m-1}}^{t_{2}}\frac{1}{\Gamma (\eta_{m})}\frac{|u\left(t_{2},s,y\left(s\right)\right)-u\left(t_{1},s,y\left(s\right)\right)|}{{\left(t_{2}-s\right)}^{1-\eta_{m}}}ds\\ & & +|\left(\Phi y\right)\left(t_{2}\right)|\frac{\left|u(t_{1},t_{1},R_{m})\right|}{\Gamma (\eta_{m}+1)}({\left(t_{2}-T_{m-1}\right)}^{\eta_{m}}-{\left(t_{2}-t_{1}\right)}^{\eta_{m}}-{\left(t_{1}-T_{m-1}\right)}^{\eta_{m}})\\ & & +|\left(\Phi y\right)\left(t_{2}\right)|\frac{\left|u(t_{2},t_{2},R_{m})\right|}{\Gamma (\eta_{m}+1)}{\left(t_{2}-t_{1}\right)}^{\eta_{m}}+\left|\left(\Phi y\right)\left(t_{2}\right)-\left(\Phi y\right)\left(t_{1}\right)\right|\frac{\left|u(t_{1},t_{1},R_{m})\right|}{\Gamma (\eta_{m}+1)}{\left(t_{1}-T_{m-1}\right)}^{\eta_{m}}. \end{array}$$

By (A4), the function u(t,s,y) is uniformly continuous on $J_{m}^{2}\times B_{R_{m}}$. Then, we have

$$\lim_{t_{2}\to t_{1}}\left|u\left(t_{2},s,y\left(s\right)\right)-u\left(t_{1},s,y\left(s\right)\right)\right|=0$$

uniformly in $s\in J_{m}$ and $y\in B_{R_{m}}$. Hence, we have

$$\left|\int_{T_{m-1}}^{t_{2}}\frac{1}{\Gamma (\eta_{m})}\frac{u\left(t_{2},s,y\left(s\right)\right)-u\left(t_{1},s,y\left(s\right)\right)}{{\left(t_{2}-s\right)}^{1-\eta_{m}}}ds\right|$$

(9) $$\leq \underset{s\in J_{m},\;y\in B_{R_{m}}}{\sup}\frac{{\left(t_{2}-T_{m-1}\right)}^{1-\eta_{m}}}{\Gamma (\eta_{m}+1)}\left|u\left(t_{2},s,y\left(s\right)\right)-u\left(t_{1},s,y\left(s\right)\right)\right|\to 0\;as\;t_{2}\to t_{1}.$$

By using the continuity of g(t) and (Φy)(t) together with (9) we conclude that $∥\left(Sy\right)\left(t_{2}\right)-\left(Sy\right)\left(t_{1}\right){∥}_{E_{m}}\to 0$ as $|t_{2}-t_{1}|\to 0$. This implies that $S(B_{R_{m}})$ is equicontinuous.

Hence, all conditions of Theorem 1 are satisfied and thus, Equation (7) has at least one solution ${\tilde{y}}_{m}\in B_{R_{m}}$. Since $B_{R_{m}}\subset E_{m}$, the assertion of Theorem 2 is proved. □

The Banach contraction principle will be used in the proof of the next result.

**Theorem** **3.** *Let the conditions of Theorem 2 be satisfied, and the inequality*

(10) $$\left(D_{m}\left(\alpha +\beta R_{m}\right)+\varphi^{⋆}K_{m}\vartheta \left(R_{m}\right)\right)\frac{\left(T_{m}^{1-\delta }-T_{m-1}^{1-\delta }\right){\left(T_{m}-T_{m-1}\right)}^{\eta_{m}-1}}{\left(1-\delta \right)\Gamma \left(\eta_{m}\right)}\leq 1$$

*holds. Then, Equation (7) has a unique solution on $E_{m}$.*

**Proof.**  We will show that $S:B_{R_{m}}\to B_{R_{m}}$ is a contraction operator.

Let $y,\bar{y}\in B_{R_{m}}$, and $t\in J_{m}$, we have

$$|\left(Sy\right)\left(t\right)-\left(S\bar{y}\right)\left(t\right)|$$

$$\begin{array}{ccc} & \leq & \left|\left(\Phi y\right)\left(t\right)\int_{T_{m-1}}^{t}\frac{1}{\Gamma (\eta_{m})}\frac{u(t,s,y(s))}{{\left(t-s\right)}^{1-\eta_{m}}}ds-\left(\Phi \bar{y}\right)\left(t\right)\int_{T_{m-1}}^{t}\frac{1}{\Gamma (\eta_{m})}\frac{u(t,s,\bar{y}\left(s\right))}{{\left(t-s\right)}^{1-\eta_{m}}}ds\right|\\ & \leq & \left|\left(\Phi y\right)\left(t\right)\int_{T_{m-1}}^{t}\frac{1}{\Gamma (\eta_{m})}\frac{u(t,s,y(s))}{{\left(t-s\right)}^{1-\eta_{m}}}ds-\left(\Phi y\right)\left(t\right)\int_{T_{m-1}}^{t}\frac{1}{\Gamma (\eta_{m})}\frac{u(t,s,\bar{y}\left(s\right))}{{\left(t-s\right)}^{1-\eta_{m}}}ds\right|\\ & + & \left|\left(\Phi y\right)\left(t\right)\int_{T_{m-1}}^{t}\frac{1}{\Gamma (\eta_{m})}\frac{u(t,s,\bar{y}\left(s\right))}{{\left(t-s\right)}^{1-\eta_{m}}}ds-\left(\Phi \bar{y}\right)\left(t\right)\int_{T_{m-1}}^{t}\frac{1}{\Gamma (\eta_{m})}\frac{u(t,s,\bar{y}\left(s\right))}{{\left(t-s\right)}^{1-\eta_{m}}}ds\right|\\ & \leq & \left|\left(\Phi y\right)\left(t\right)\right|\int_{T_{m-1}}^{t}\frac{1}{\Gamma (\eta_{m})}\frac{1}{{\left(t-s\right)}^{1-\eta_{m}}}\left|u\left(t,s,y\left(s\right)\right)ds-u\left(t,s,\bar{y}\left(s\right)\right)\right|ds\\ & + & |\left(\Phi y\right)\left(t\right)-\left(\Phi \bar{y}\right)\left(t\right)|\int_{T_{m-1}}^{t}\frac{1}{\Gamma (\eta_{m})}\frac{\left|u(t,s,\tilde{y}\left(s\right))\right|}{{\left(t-s\right)}^{1-\eta_{m}}}ds\\ & \leq & \left(\alpha +\beta |y\left(t\right)|\right)D_{m}\int_{T_{m-1}}^{t}\frac{1}{\Gamma (\eta_{m})}\frac{1}{{\left(t-s\right)}^{1-\eta_{m}}}s^{-\delta }\left|y\left(s\right)-\bar{y}\left(s\right)\right|ds\\ & + & K_{m}|y\left(s\right)-\bar{y}\left(s\right)|\int_{T_{m-1}}^{t}\frac{1}{\Gamma (\eta_{m})}\frac{1}{{\left(t-s\right)}^{1-\eta_{m}}}s^{-\delta }\varphi \left(s\right)\vartheta (|\bar{y}\left(s\right)\left|\right)ds\\ & \leq & [(D_{m}\left(\alpha +\beta R_{m}\right)+\varphi^{⋆}K_{m}\vartheta \left(R_{m}\right))\frac{\left(T_{m}^{1-\delta }-T_{m-1}^{1-\delta }\right){\left(T_{m}-T_{m-1}\right)}^{\eta_{m}-1}}{\left(1-\delta \right)\Gamma \left(\eta_{m}\right)}]{∥y-\bar{y}∥}_{E_{m}}. \end{array}$$

Therefore,

$$∥\left(Sy\right)\left(t\right)-\left(S\bar{y}\right)\left(t\right){∥}_{E_{m}}\leq [(D_{m}\left(\alpha +\beta R_{m}\right)+\varphi^{⋆}K_{m}\vartheta \left(R_{m}\right))\frac{\left(T_{m}^{1-\delta }-T_{m-1}^{1-\delta }\right){\left(T_{m}-T_{m-1}\right)}^{\eta_{m}-1}}{\left(1-\delta \right)\Gamma \left(\eta_{m}\right)}]{∥y-\bar{y}∥}_{E_{m}}.$$

Ergo, by (10), we conclude that the operator *S* composes a contraction. Therefore, by the Banach’s contraction principle, *S* has a unique fixed point ${\tilde{y}}_{m}$ in $B_{R_{m}}\subset E_{m}$, which is the unique solution of Equation (7). This proves Theorem 2. □

The existence result for Equation (2) will be proved in the next theorem.

**Theorem** **4.** *Suppose that assumptions (A1)–(A5) and inequalities (8), (10) are satisfied for all m∈{1,2,…,n}.*

*Then, Equation (2) has a unique solution in C(J,R).*

**Proof.**  According to Theorem 3 the fractional integral equation of a constant order (7) has a unique solution ${\tilde{y}}_{m}\in E_{m}$ for any m∈{1,2,…,n}. We construct the function

(11) $$y_{m}=\left\{\begin{array}{c} 0,\;\;\;t\in [0,T_{m-1}],\\ {\tilde{y}}_{m},\;\;\;t\in J_{m} \end{array}\right.$$

defined for m∈{1,2,…,n}. It is clear that $y_{m}\in C\left(\left[0,T_{m}\right],R\right)$ is a solution of the integral Equation (6) for $t\in J_{m}.$ Then, the function

$$y\left(t\right)=\left\{\begin{array}{c} y_{1}\left(t\right),\;\;\;\;t\in J_{1},\\ y_{2}\left(t\right)=\left\{\begin{array}{c} 0,\;\;\;\;t\in J_{1},\\ {\tilde{y}}_{2},\;\;\;t\in J_{2} \end{array}\right.\;\;\;.\;\;\;\;\;\;\;\\ \;\;\;.\;\;\;\;\;\;\;\\ \;\;\;.\;\;\;\;\;\;\;\\ \;\;\;.\;\;\;\;\;\;\;\\ y_{n}\left(t\right)=\left\{\begin{array}{c} 0,\;\;\;\;t\in [0,T_{n-1}],\\ {\tilde{y}}_{n},\;\;\;t\in J_{n} \end{array}\right. \end{array}\right.$$

is the unique solution of Equation (2) in C(J,R). □

**Remark** **2.** *Since the quadratic integral equations of the Urysohn type are widely used in theory and applications, the proposed generalization extends the opportunities for its application. In addition, the established existence and uniqueness results open the door for the study of the qualitative properties of these types of equations such as stability, periodicity, asymptotic behavior, etc.*

**Remark** **3.** *One of the states that is of great importance to researchers of the Urysohn type and related models are the so called “steady” or equilibrium states. Another solution of interest to applied sciences is the periodic solution. The results proposed in this paper can be applied to such specific solutions of interest. Hence, in the case, when y(t) is one of the states of interest, the providing results can be used so by means of convenient existence and uniqueness results to guarantee their fundamental properties.*

**Remark** **4.** *For η(t)=1, t∈J our existence and uniqueness results are consistent with the results for integer-order quadratic integral equations of the Urysohn type [5,9]. Thus, our results extend some existing results. Moreover, since fractional variable-order derivatives are more general than the integer-order once, as well as than the fractional derivatives of constant order, the proposed model and results have a wider applicability and can better reflect the dependence process on the historical information. In addition, the bigger degree of freedom can make the theory of this important class of equation more consistent with the numerical simulations.*

## 4. Ulam–Hyers Stability

**Theorem** **5.** *Let the conditions of Theorem 4 be satisfied, for all m∈{1,2,…,n}. Then, the integral Equation (2) is Ulam–Hyers stable*

**Proof.**  Let ϵ>0 be an arbitrary number and the function z(t) from C(J,R) satisfy the inequality (4)

For m∈{1,2,…,n}, we define the functions $z_{1}\left(t\right)\equiv z\left(t\right),\;t\in \left[0,T_{1}\right]$ and for m=2,3,…,n:

$$z_{m}\left(t\right)=\left\{\begin{array}{c} 0,\;\;\;\;t\in [0,T_{m-1}],\;\\ z\left(t\right),\;\;\;\;t\in J_{m}. \end{array}\right.$$

According to Theorem 4, the integral Equation (2) has a unique solution y∈C(J,R) defined by $y\left(t\right)=y_{m}\left(t\right)$ for $t\in J_{m},\;m=1,2,\ldots ,n$, where

(12) $$y_{m}=\left\{\begin{array}{c} 0,\;\;\;t\in [0,T_{m-1}],\\ {\tilde{y}}_{m},\;\;\;t\in J_{m}, \end{array}\right.$$

and ${\tilde{y}}_{m}\in E_{m}$ is a unique solution of the integral Equation (7)

Let $t\in J_{m},\;m=1,2,\ldots ,n$. Then, we obtain

$$\begin{array}{ccc} & & |z\left(t\right)-y\left(t\right)|=|z\left(t\right)-y_{m}\left(t\right)|=|z_{m}\left(t\right)-{\tilde{y}}_{m}\left(t\right)|\\ & = & \left|z_{m}\left(t\right)-g\left(t\right)-\left(\Phi {\tilde{y}}_{m}\right)\left(t\right)\int_{T_{m-1}}^{t}\frac{1}{\Gamma (\eta_{m})}\frac{u(t,s,{\tilde{y}}_{m}\left(s\right))}{{\left(t-s\right)}^{1-\eta_{m}}}ds\right|\\ & \leq & \left|z_{m}\left(t\right)-g\left(t\right)-(\Phi z_{m}\left(t\right)\int_{T_{m-1}}^{t}\frac{1}{\Gamma (\eta_{m})}\frac{u(t,s,z_{m}\left(s\right))}{{\left(t-s\right)}^{1-\eta_{m}}}ds\right|\\ & + & \left|\left(\Phi z_{m}\right)\left(t\right)\int_{T_{m-1}}^{t}\frac{1}{\Gamma (\eta_{m})}\frac{u(t,s,z_{m}\left(s\right))}{{\left(t-s\right)}^{1-\eta_{m}}}ds-\left(\Phi {\tilde{y}}_{m}\right)\left(t\right)\int_{T_{m-1}}^{t}\frac{1}{\Gamma (\eta_{m})}\frac{u(t,s,{\tilde{y}}_{m}\left(s\right))}{{\left(t-s\right)}^{1-\eta_{m}}}ds\right|\\ & \leq & \epsilon +|\left(\Phi z_{m}\right)\left(t\right)\int_{T_{m-1}}^{t}\frac{1}{\Gamma (\eta_{m})}\frac{u(t,s,z_{m}\left(s\right))}{{\left(t-s\right)}^{1-\eta_{m}}}ds-\left(\Phi {\tilde{y}}_{m}\right)\left(t\right)\int_{T_{m-1}}^{t}\frac{1}{\Gamma (\eta_{m})}\frac{u(t,s,{\tilde{y}}_{m}\left(s\right))}{{\left(t-s\right)}^{1-\eta_{m}}}ds|\\ & \leq & \epsilon +|\left(\Phi z_{m}\right)\left(t\right)\int_{T_{m-1}}^{t}\frac{1}{\Gamma (\eta_{m})}\frac{u(t,s,z_{m}\left(s\right))}{{\left(t-s\right)}^{1-\eta_{m}}}ds-\left(\Phi z_{m}\right)\left(t\right)\int_{T_{m-1}}^{t}\frac{1}{\Gamma (\eta_{m})}\frac{u(t,s,{\tilde{y}}_{m}\left(s\right))}{{\left(t-s\right)}^{1-\eta_{m}}}ds|\\ & + & \left|\left(\Phi z_{m}\right)\left(t\right)\int_{T_{m-1}}^{t}\frac{1}{\Gamma (\eta_{m})}\frac{u(t,s,{\tilde{y}}_{m}\left(s\right))}{{\left(t-s\right)}^{1-\eta_{m}}}ds-\left(\Phi {\tilde{y}}_{m}\right)\left(t\right)\int_{T_{m-1}}^{t}\frac{1}{\Gamma (\eta_{m})}\frac{u(t,s,{\tilde{y}}_{m}\left(s\right))}{{\left(t-s\right)}^{1-\eta_{m}}}ds\right|\\ & \leq & \epsilon +|\left(\Phi z_{m}\right)\left(t\right)|\int_{T_{m-1}}^{t}\frac{1}{\Gamma (\eta_{m})}\frac{1}{{\left(t-s\right)}^{1-\eta_{m}}}\left|u\left(t,s,z_{m}\left(s\right)\right)ds-u\left(t,s,{\tilde{y}}_{m}\left(s\right)\right)\right|ds\\ & + & |\left(\Phi z_{m}\right)\left(t\right)-\left(\Phi {\tilde{y}}_{m}\right)\left(t\right)|\int_{T_{m-1}}^{t}\frac{1}{\Gamma (\eta_{m})}\frac{\left|u(t,s,\tilde{y}\left(s\right))\right|}{{\left(t-s\right)}^{1-\eta_{m}}}ds\\ & \leq & \epsilon +(\alpha +\beta |z_{m}\left(t\right)|)D_{m}\int_{T_{m-1}}^{t}\frac{1}{\Gamma (\eta_{m})}\frac{1}{{\left(t-s\right)}^{1-\eta_{m}}}s^{-\delta }\left|z_{m}\left(s\right)-{\tilde{y}}_{m}\left(s\right)\right|ds\\ & + & K_{m}|z_{m}\left(s\right)-{\tilde{y}}_{m}\left(s\right)|\int_{T_{m-1}}^{t}\frac{1}{\Gamma (\eta_{m})}\frac{1}{{\left(t-s\right)}^{1-\eta_{m}}}s^{-\delta }\varphi \left(s\right)\vartheta (|{\tilde{y}}_{m}\left(s\right)\left|\right)ds\\ & \leq & \epsilon +[(D_{m}\left(\alpha +\beta R_{m}\right)+\varphi^{⋆}K_{m}\vartheta \left(R_{m}\right))\frac{\left(T_{m}^{1-\delta }-T_{m-1}^{1-\delta }\right){\left(T_{m}-T_{m-1}\right)}^{\eta_{m}-1}}{\left(1-\delta \right)\Gamma \left(\eta_{m}\right)}]{∥z_{m}-{\tilde{y}}_{m}∥}_{E_{m}}. \end{array}$$

Then,

$$\begin{array}{c} ∥z-y∥\left(1-\left[(D_{m}\left(\alpha +\beta R_{m}\right)+\varphi^{⋆}K_{m}\vartheta \left(R_{m}\right))\frac{\left(T_{m}^{1-\delta }-T_{m-1}^{1-\delta }\right){\left(T_{m}-T_{m-1}\right)}^{\eta_{m}-1}}{\left(1-\delta \right)\Gamma \left(\eta_{m}\right)}\right]\right)\leq \epsilon . \end{array}$$

Thus, for each $t\in J_{m}$ we obtain

$$\begin{array}{ccc} \left|z(t)-y(t)\right| & \leq & ∥z-y∥\\ & \leq & \frac{1}{\left(1-\left[(D_{m}\left(\alpha +\beta R_{m}\right)+\varphi^{⋆}K_{m}\vartheta \left(R_{m}\right))\frac{\left(T_{m}^{1-\delta }-T_{m-1}^{1-\delta }\right){\left(T_{m}-T_{m-1}\right)}^{\eta_{m}-1}}{\left(1-\delta \right)\Gamma \left(\eta_{m}\right)}\right]\right)}\epsilon :=c_{u}\epsilon . \end{array}$$

Therefore, the integral Equation (2) is Ulam–Hyers stable. □

## 5. An Example

**Example** **1.** *In this example, we deal with the following quadratic integral equation of the Urysohn type of fractional variable order*

(13) $$y\left(t\right)=\frac{1}{t+2}+\frac{\left|y(t)\right|}{2+|y(t)|}\int_{0}^{t}\frac{1}{\Gamma (\eta (t))}.\frac{t^{-\frac{1}{10}}}{t+2}.\frac{y(s)}{s+10}.\frac{1}{{\left(t-s\right)}^{1-\eta (t)}}ds,\;\;\;t\in J:=\left[0,2\right],$$

*where*

(14) $$\eta \left(t\right)=\left\{\begin{array}{c} \frac{3}{2},\;\;\;\;t\in J_{1}:=\left[0,1\right],\\ \frac{6}{5},\;\;\;\;t\in J_{2}:=]1,2]. \end{array}\right.$$

*Let*

$$T_{0}=0,T_{1}=1,T_{2}=T=2,$$

$$g\left(t\right)=\frac{1}{t+2},\;\;\;\;t\in J,$$

$$\left(\Phi y\right)\left(t\right)=\frac{y(t)}{2+y(t)},\;\;\;\;t\in J\;\;\;\;and\;\;\;\;y\in C\left(J,R_{+}\right),$$

$$u\left(t,s,y\right)=\frac{t^{-\frac{1}{10}}}{t+2}.\frac{1}{s+10}.y,\;\left(t,s,y\right)\in J^{2}\times R_{+},$$

$$\;and\;y\in C(J,R_{+}).$$

*By (14), according to (7) we consider the following two auxiliary equations*

(15) $$y\left(t\right)=\frac{1}{t+2}+\frac{\left|y(t)\right|}{2+|y(t)|}\int_{0}^{t}\frac{1}{\Gamma (\eta_{1})}.\frac{t^{-\frac{1}{10}}}{t+2}.\frac{y(s)}{s+10}.\frac{1}{{\left(t-s\right)}^{1-\eta_{1}}}ds,\;\;\;t\in J_{1}$$

*and*

(16) $$y\left(t\right)=\frac{1}{t+2}+\frac{\left|y(t)\right|}{2+|y(t)|}\int_{0}^{t}\frac{1}{\Gamma (\eta_{2})}.\frac{t^{-\frac{1}{10}}}{t+2}.\frac{y(s)}{s+10}.\frac{1}{{\left(t-s\right)}^{1-\eta_{2}}}ds,\;\;\;t\in J_{2}.$$

*We will show that assumptions (A1)–(A5) and inequalities (8), (10) hold.*

*For t∈J, we have*

$$|\left(\Phi y\right)\left(t\right)-\left(\Phi \bar{y}\right)\left(t\right)\left|=\right|\frac{y(t)}{2+y(t)}-\frac{\bar{y}\left(t\right)}{2+\bar{y}\left(t\right)}\left|=\right|\frac{2y\left(t\right)-2\bar{y}\left(t\right)}{\left(2+y\left(t\right)\right)\left(2+\bar{y}\left(t\right)\right)}|\leq 2|y\left(t\right)-\bar{y}\left(t\right)|.$$

*Then, (A2) is satisfied with $K_{1}=2$ and each $y,\bar{y}\in E_{1}$. Moreover,*

$$\left|\left(\Phi y\right)\left(t\right)|=\right|\frac{y(t)}{2+y(t)}|\leq |y\left(t\right)|.$$

*Then, assumption (A3) holds for α=0 and β=1 and each $y\in E_{1}$. Calculate*

$$t^{\frac{1}{10}}|u\left(t,s,y\right)-u\left(t,s,\bar{y}\right)\left|=\right|\frac{1}{t+2}.\frac{1}{s+10}.y-\frac{1}{t+2}.\frac{1}{s+10}.\bar{y}|=\frac{1}{t+2}.\frac{1}{s+10}|y-\bar{y}|\leq \frac{1}{20}\left|y-\bar{y}\right|.$$

*Hence, assumption (A4) is satisfied with $\delta =\frac{1}{10}$ and $D_{1}=\frac{1}{20}$ for all $\left(t,s\right)\in J_{1}^{2}$ and $y,\bar{y}\in E_{1}$.*

*We have that*

$$t^{\frac{1}{10}}\left|u\left(t,s,y\right)\right|=\frac{1}{t+2}.\frac{1}{s+10}.|y|\leq \varphi \left(s\right)\vartheta \left(|y|\right).$$

*Then, (A5) holds with $\delta =\frac{1}{10}$, ϑ(|y|)=|y| and $\varphi \left(s\right)=\frac{1}{2(s+10)}$, which means that $\varphi^{⋆}=\frac{1}{20}$ and the inequality*

$$\frac{R_{1}}{g^{⋆}+\frac{\left(T_{1}^{1-\delta }-T_{0}^{1-\delta }\right){\left(T_{1}-T_{0}\right)}^{\eta_{1}-1}}{\left(1-\delta \right)\Gamma \left(\eta_{1}\right)}\left(\alpha +\beta R_{1}\right)\vartheta \left(R_{1}\right)\varphi^{⋆}}>1$$

*is satisfied for each* $R_{1}\in \left(0.5175,\;15.4568\right)$ *. Hence, condition (8) holds and the inequality*

$$\left(D_{1}\left(\alpha +\beta R_{1}\right)+\varphi^{⋆}K_{1}\vartheta \left(R_{1}\right)\right)\frac{\left(T_{1}^{1-\delta }-T_{0}^{1-\delta }\right){\left(T_{1}-T_{0}\right)}^{\eta_{1}-1}}{\left(1-\delta \right)\Gamma \left(\eta_{1}\right)}\leq 1$$

*is satisfied for each $R_{1}\in \left(0,5.3172\right)$ which means that condition (10) holds.*

*It follows from Theorem 3 that the Equation (15) has a unique solution $\tilde{y_{1}}$ in $E_{1}$.*

*For t∈J, we have*

$$|\left(\Phi y\right)\left(t\right)-\left(\Phi \bar{y}\right)\left(t\right)\left|=\right|\frac{y(t)}{2+y(t)}-\frac{\bar{y}\left(t\right)}{2+\bar{y}\left(t\right)}\left|=\right|\frac{2y\left(t\right)-2\bar{y}\left(t\right)}{\left(2+y\left(t\right)\right)\left(2+\bar{y}\left(t\right)\right)}|\leq 2|y\left(t\right)-\bar{y}\left(t\right)|.$$

*Then, (A2) is satisfied with $K_{2}=2$ and for each $y,\bar{y}\in E_{2}$. Moreover,*

$$\left|\left(\Phi y\right)\left(t\right)|=\right|\frac{y(t)}{2+y(t)}|\leq |y\left(t\right)|.$$

*Then, (A3) holds with α=0 and β=1 and for each $y\in E_{2}$. We have that*

$$t^{\frac{1}{10}}|u\left(t,s,y\right)-u\left(t,s,\bar{y}\right)\left|=\right|\frac{1}{t+2}.\frac{1}{s+10}.y-\frac{1}{t+2}.\frac{1}{s+10}.\bar{y}|=\frac{1}{t+2}.\frac{1}{s+10}|y-\bar{y}|\leq \frac{1}{33}\left|y-\bar{y}\right|.$$

*Hence, (A4) is satisfied with $\delta =\frac{1}{10}$ and $D_{2}=\frac{1}{33}$ for all $\left(t,s\right)\in J_{2}^{2}$ and $y,\bar{y}\in E_{2}$. In addition, we have*

$$t^{\frac{1}{10}}\left|u\left(t,s,y\right)\right|=\frac{1}{t+2}.\frac{1}{s+10}.|y|\leq \varphi \left(s\right)\vartheta \left(|y|\right).$$

*Then, (H5) holds with $\delta =\frac{1}{10}$, ϑ(|y|)=|y| and $\varphi \left(s\right)=\frac{1}{3(s+10)}$ which means that $\varphi^{⋆}=\frac{1}{33}$ and the inequality*

$$\frac{R_{2}}{g^{⋆}+\frac{\left(T_{2}^{1-\delta }-T_{1}^{1-\delta }\right){\left(T_{2}-T_{1}\right)}^{\eta_{2}-1}}{\left(1-\delta \right)\Gamma \left(\eta_{2}\right)}\left(\alpha +\beta R_{2}\right)\vartheta \left(R_{2}\right)\varphi^{⋆}}>1$$

*is satisfied for each $R_{2}\in \left(0.6687,\;30.8769\right)$. Thus, condition (8) holds and the inequality*

$$\left(D_{2}\left(\alpha +\beta R_{2}\right)+\varphi^{⋆}K_{2}\vartheta \left(R_{2}\right)\right)\frac{\left(T_{2}^{1-\delta }-T_{1}^{1-\delta }\right){\left(T_{2}-T_{1}\right)}^{\eta_{2}-1}}{\left(1-\delta \right)\Gamma \left(\eta_{2}\right)}\leq 1$$

*is satisfied for each $R_{2}\in \left(0,\;10.4948\right)$. Hence, condition (10) holds. Consequently, from Theorem 3, Equation (16) has a unique solution $\tilde{y_{2}}$ in $E_{2}$.*

*Then, by Theorem 4, the Equation (13) has a unique solution*

$$y\left(t\right)=\left\{\begin{array}{c} \tilde{y_{1}}\left(t\right),\;\;\;\;t\in J_{1},\\ y_{2}\left(t\right),\;\;\;\;t\in J_{2}, \end{array}\right.$$

*where*

$$y_{2}=\left\{\begin{array}{c} 0,\;\;\;t\in [0,T_{1}],\\ \tilde{y_{2}},\;\;\;t\in J_{2}. \end{array}\right.$$

*In addition, according to Theorem 5, the integral Equation (13) is Ulam–Hyers stable.*

**Remark** **5.** *The proposed example shows the feasibility of our fundamental results. Since the obtained criteria are in the form of algebraic inequalities, they can be easily applied.*

**Remark** **6.** *Example 1 also demonstrated that the proposed existence and uniqueness results can be used in the study of the qualitative properties of the solutions of the introduced model of fractional variable order.*

## 6. Conclusions

In this paper, we present results about the existence and uniqueness of solutions for a quadratic integral equation of the Urysohn type of fractional variable order η(t), where η(t):[0,T]→(1,2] is a piece-wise constant function. All our results are based on the Schauder’s fixed-point theorem and the Banach contraction principle. The theoretical findings are also illustrated by an numerical example. Since fractional integral operators of variable order are applied in different models of real-world phenomena, we expect that the proposed results will be of interest to numerous audiences of researchers in mathematics, engineering and applied sciences. Moreover, the outcome of this research paper will benefit the investigations on integral equations of the Urysohn type in other spaces such as Frechet space. Furthermore, one could study the proposed integral equation with different fractional integrals, such as Caputo type, Hadamard type, Hilfer type and some others. It is possible to extend the results to the uncertain case since a real system always involves uncertainties due to some disturbances in system, inaccuracy in model parameter measurements or noises from external inputs, and the analysis of models with uncertainties is essential for theory and applications. A future direction of our investigations is also related to the study of the impact of continuous and impulsive controllers on the qualitative behavior of the introduced model.

## Data Availability

Not applicable.
